# Neurooncology: 2026 update

**DOI:** 10.17879/freeneuropathology-2026-9688

**Published:** 2026-07-09

**Authors:** Michel Mittelbronn

**Affiliations:** 1 Division of Neuropathology, Department of Pathology and Neuropathology, Medical Faculty, University of Cologne, Germany; 2 Department of Health, Medicine and Life Sciences (DHML); University of Luxembourg; Esch sur Alzette, Luxembourg; 3 Faculty of Science, Technology and Medicine (FSTM), University of Luxembourg, Esch-sur-Alzette, Luxembourg

**Keywords:** Neurooncology, Neuropathology, Brain tumors, Glioblastoma, Brain metastasis

## Abstract

The present collection of studies highlights major conceptual and translational advances in contemporary neurooncology related to the field of neuropathology. Central themes include the increasing recognition of neuron-tumor interactions, immune microenvironment remodeling, vascular heterogeneity, and epigenetic plasticity as key drivers of brain tumor progression and therapeutic resistance. Glioblastoma emerges as a highly dynamic and synaptically integrated disease entity, while melanoma brain metastases demonstrate profound microglial reprogramming with direct implications for immunotherapy. Novel molecular and spatial profiling approaches further reveal distinct vascular and immune landscapes across gliomas and brain metastases as well as lineage-dependent developmental programs in medulloblastoma. In parallel, rapid advances in artificial intelligence, nanopore sequencing, and real-time molecular diagnostics are reshaping neuropathological workflows and intraoperative decision-making. Proteomic and multi-omics analyses additionally uncover clinically relevant immune-hot glioma subtypes associated with adverse prognosis and spatial immune remodeling. Together, these studies underscore the increasing convergence of molecular neurobiology, immunology, epigenetics, and computational pathology in modern neurooncology and highlight emerging opportunities for precision diagnostics and targeted therapeutic intervention.

## Introduction

 In 2025, the landscape of global health has been highly complex and unforgiving. Nations continue to recover from the socioeconomic fallout of successive pandemics, healthcare systems are strained by demographic shifts toward older populations, and inequities in access to advanced medical care have widened between highincome and resourcelimited regions. Over the brief span from 2019 to 2021, global life expectancy plunged by 1.8 years — the sharpest decline in modern records — wiping out a decade of accumulated health progress **[World health statistics, 2025]**. Against this backdrop, neurooncology stands at a precarious crossroads. Cancers of the brain and spinal cord have long defied the progress that has characterized other oncological arenas and incidence rates for malignant central nervous system (CNS) tumors are projected to increase by more than 30 % until 2050 **[Kim et al., 2025]**. While survival rates for many solid tumors have improved incrementally or dramatically over the past decade, neurooncological diseases have remained stubbornly lethal. Glioblastoma, diffuse intrinsic pontine glioma (DIPG), and metastatic brain tumors remain associated with devastating morbidity and mortality, contributing to severe neurologic impairment, loss of quality of life, and significant long-term care requirements. CNS malignancies persist as a leading cause of cancerrelated mortality in children and young adults, and an increasingly prevalent burden in aging populations worldwide. Of note, only one third of adult glioblastoma patients present with a condition allowing for an aggressive multimodal treatment even in higher developed countries **[Le Calvez et al., 2025]**. This is associated with a median overall survival of far below one year in glioblastoma patients in population-based analyses while most publications from quaternary care centers present median survival rates going beyond one year **[Pack et al., 2026]**. At the same time, the field is entering a period of remarkable scientific acceleration. Advances in tumor immunology, spatial and single-cell profiling, artificial intelligence (AI), rapid intraoperative diagnostics, and patient derived modeling systems are beginning to reshape how neurooncological diseases are understood and treated. The following selection of papers reflects these converging developments, highlighting studies that not only deepen biological insight into CNS malignancies, but also point toward more precise, adaptive, and clinically translatable neuropathological approaches for the next generation of neurooncological care. 

**With this, the "top ten" series in neurooncology for 2025 reads as follows:**

1. Rapid synaptic integration of glioblastoma into neural circuits drives invasion and confers therapeutic resistance [Tetzlaff et al., 2025]
2. Reprogramming microglia to enhance antitumoral immunity in melanoma brain metastasis [Rodriguez-Baena et al., 2025]
3. Divergent vascular and immune landscapes in gliomas and brain metastases [Bejarano et al., 2025]
4. Context-dependent pro-tumorigenic functions of ZIC1 in medulloblastoma [Lee et al., 2025]
5. Prognostic immune-hot proteomic subtypes in IDH-mutant glioma [Tang et al., 2025]
6. Opening the black box of brain tumor AI diagnostics [Benfatto et al., 2025]
7. Real-time epigenetic brain tumor classification with sparse nanopore data [Brändl et al., 2025]
8. Nanopore sequencing enters the operating room: rapid molecular profiling of CNS tumors [Patel et al., 2025]
9. Real-time AI-guided detection of glioma surgical margins using label-free optical microscopy and a self-supervised foundation model [Kondepudi et al., 2025]
10. Patient-derived brain tumor organoids: toward functional precision neurooncology [Peng et al., 2025]

## 1. Rapid synaptic integration of glioblastoma into neural circuits drives invasion and confers therapeutic resistance [Tetzlaff et al., 2025]

**Figure 1: Schematic representation of neuron-glioma interactions and experimental targeting strategies:**
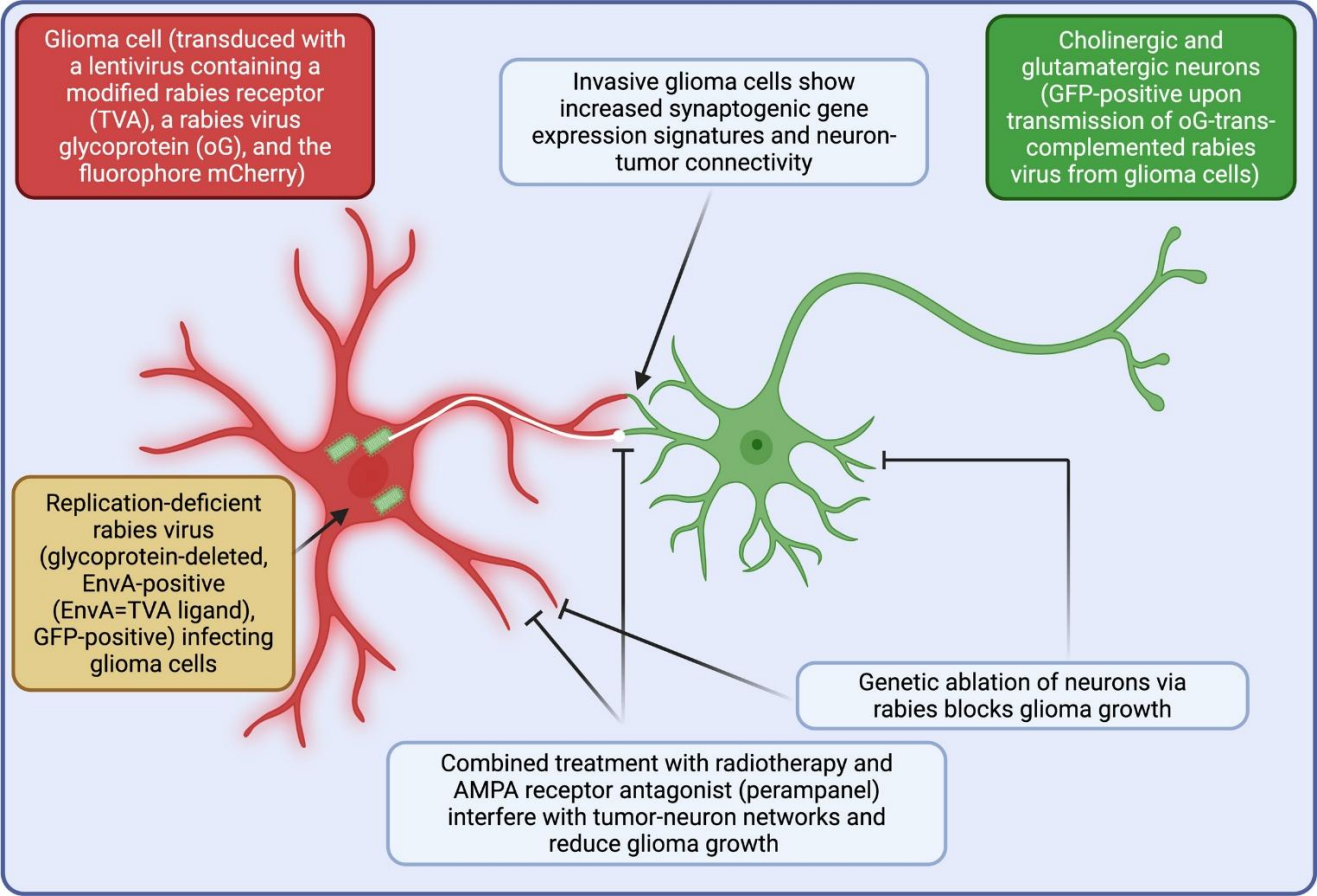


Tetzlaff et al. examine how glioblastoma integrates into neuronal circuits and whether these neuron-tumor networks can be therapeutically targeted **[Tetzlaff et al., 2025]**. The authors repurpose monosynaptic retrograde rabies tracing, engineering "starter" glioma cells to express an entry receptor (TVA) for a modified rabies, rabies glycoprotein (oG), and the fluorophore mCherry tracer so that a modified glycoproteindeleted, GFP (green fluorescent protein)-positive rabies virus expressing the TVA-ligand EnvA exclusively infects TVA-positive glioma cells subsequently — after trans-complementation with oG glycoprotein — labeling neurons exhibiting direct synaptic contacts onto tumor cells **(Figure 1)**. This enables a brainwide, monosynaptic map of neuron-tumor connectivity. Using this framework, they show that glioma cells rapidly acquire extensive synaptic input from distributed cortical and subcortical neurons, with inputtostarter ratios increasing as tumors grow. Neurons presynaptic to the tumor exhibit altered activity patterns and greater network synchronization than unconnected neurons, and imaging reveals dendritic remodeling and spine changes in connected regions. Thus, the tumor forms a dynamic, bidirectional network with the brain, simultaneously receiving synaptic drive and reshaping neuronal structure and function. The study identifies which neurons and tumor cell states underpin this connectivity. By combining tracing with singlecell and spatial transcriptomics and neurotransmitter typing, the authors show that specific neuronal subtypes — particularly cholinergic neurons — are prominently represented among tumor connected cells, and confirm cholinergic synapses onto glioma cells in experimental models and human tissue. On the tumor side, they define "synaptogenic" cell states with high expression of synapserelated gene modules, which correlate with stronger connectivity and greater invasiveness. Integration of methylation data from a clinical cohort and public multiomics datasets indicates that these synaptogenic programs are epigenetically regulated and associate with patient outcome, linking connectivityrelated states to prognosis. Mechanistically, acetylcholine signaling via the muscarinic receptor CHRM3 emerges as a key pathway. In cocultures, cholinergic stimulation enhances glioblastoma proliferation and motility, while CHRM3 knockdown reduces tumor growth *in vivo*, indicating that glioblastoma exploits canonical neurotransmitter receptors to translate neuronal activity into tumorpromoting signals. The authors also address standard therapy by showing that radiotherapy increases neuronal activity and enhances neuron-tumor connectivity, suggesting a dual effect in which irradiation damages tumor cells but concurrently augments the synaptic input that can support their survival and invasion. To counter this, they combine radiotherapy with perampanel, an AMPA receptor antagonist that dampens excitatory transmission. *In vitro* and *in vivo*, this combination blunts activitydependent tumor growth and yields better tumor control and survival than either treatment alone, demonstrating that modulating neuronal activity can improve the efficacy of conventional therapy. Finally, using rabiesbased genetic tools, the authors selectively ablate connectedTUM neurons by inducing apoptosis specifically in retrogradely labeled neurons. This targeted ablation halts glioblastoma progression and prolongs survival in preclinical models, with acceptable functional tolerance in the observation period, providing strong causal evidence that neuron-tumor networks are necessary for full malignant behavior. Overall, the paper reframes glioblastoma as a synaptically integrated component of brain circuitry. It shows that specific neuronal subtypes and synaptogenic, epigenetically defined tumor states drive functional neuron-tumor connectivity; that this connectivity is exploited via conventional neurotransmitter pathways, amplified by radiotherapyinduced hyperexcitability; and that both pharmacologic neuromodulation and targeted disruption of tumorconnected neurons can significantly restrain tumor growth. These findings support neuron-tumor networks as actionable therapeutic targets and argue for integrating circuitlevel interventions with standard glioblastoma treatments. Surprisingly, previous data from the same and other research teams presented microtubes as the key connection between neurons and glioma cells **[Venkataramani et al., 2019; Venkatesh et al., 2019]**; however, those structures are not prominently addressed in the present study. Therefore, the central question about the key glioma-neuron interaction routes remains.

## 2. Reprogramming microglia to enhance antitumoral immunity in melanoma brain metastasis [Rodriguez-Baena et al., 2025]

Melanoma brain metastases (MBM) remain associated with poor prognosis despite considerable advances in immune checkpoint inhibition. Increasing evidence suggests that the cerebral microenvironment critically contributes to metastatic progression and therapeutic resistance. Previous studies revealed that microglial cells play an especially important role in the suppression of brain metastasis formation **[Evans et al., 2023]**. In this highly elegant study, Rodriguez-Baena et al. investigated the role of microglia in MBM using syngeneic mouse models, single-cell transcriptomics and genetic as well as pharmacological targeting approaches **[Rodriguez-Baena et al., 2025]**. Interestingly, depletion experiments revealed a dual and time-dependent role of these cells. Early depletion prior to metastatic colonization increased metastatic burden, indicating an initial protective and antitumoral microglial function. In contrast, depletion after metastatic establishment reduced MBM growth, thereby suggesting that microglia undergo a tumor-associated phenotypic switch during disease progression. Transcriptomic analyses identified activation of the RELA/NF-kB pathway as a central mechanism underlying this transition. Tumor-associated microglia displayed reduced expression of canonical homeostatic markers such as TMEM119 and P2RY12 together with increased NF-kB-associated transcriptional programs. Importantly, RELA activation was also validated in human MBM samples, underscoring the translational relevance of the findings. A major strength of the study is the combination of genetic and pharmacological intervention strategies. Conditional deletion of *Rela* in CX3CR1-positive cells significantly reduced metastatic burden and prolonged survival in murine MBM models. Similar effects were achieved using the blood-brain barrier-penetrating NF-kB inhibitor dehydroxymethylepoxyquinomicin (DHMEQ). Notably, these effects were largely restricted to cerebral metastases, whereas extracranial melanoma growth remained unaffected, highlighting the unique biological role of brain-resident macrophages. Single-cell analyses further revealed that NF-kB inhibition induced a microglial reprogramming toward a proinflammatory phenotype characterized by increased expression of chemokines such as CCL4, CCL5 and CXCL10 together with enhanced antigen presentation machinery. This shift was associated with increased infiltration of CD8-positive T cells and NK cells into MBM and enhanced responsiveness to immune checkpoint inhibition.

Despite the outstanding quality of the work, some limitations should be considered. The study relies predominantly on murine models that cannot fully recapitulate the heterogeneity of human MBM. In addition, CX3CR1-based targeting is not entirely microglia-specific and may partially affect peripheral myeloid populations. Furthermore, systemic NF-kB inhibition may potentially induce broader immunological side effects in clinical settings. Nevertheless, Rodriguez-Baena et al. provide one of the most comprehensive analyses of microglial plasticity in melanoma brain metastasis to date. The study significantly advances the understanding of the cerebral immune microenvironment and identifies microglial NF-kB signaling as a promising therapeutic target capable of enhancing antitumoral immunity and improving responses to immunotherapy in MBM **(Figure 2)**.

**Figure 2: Microglial reprogramming to proinflammatory (PIM) and homeostatic-like (HM) phenotypes via NF-kB inhibition reduces melanoma brain metastasis progression:**
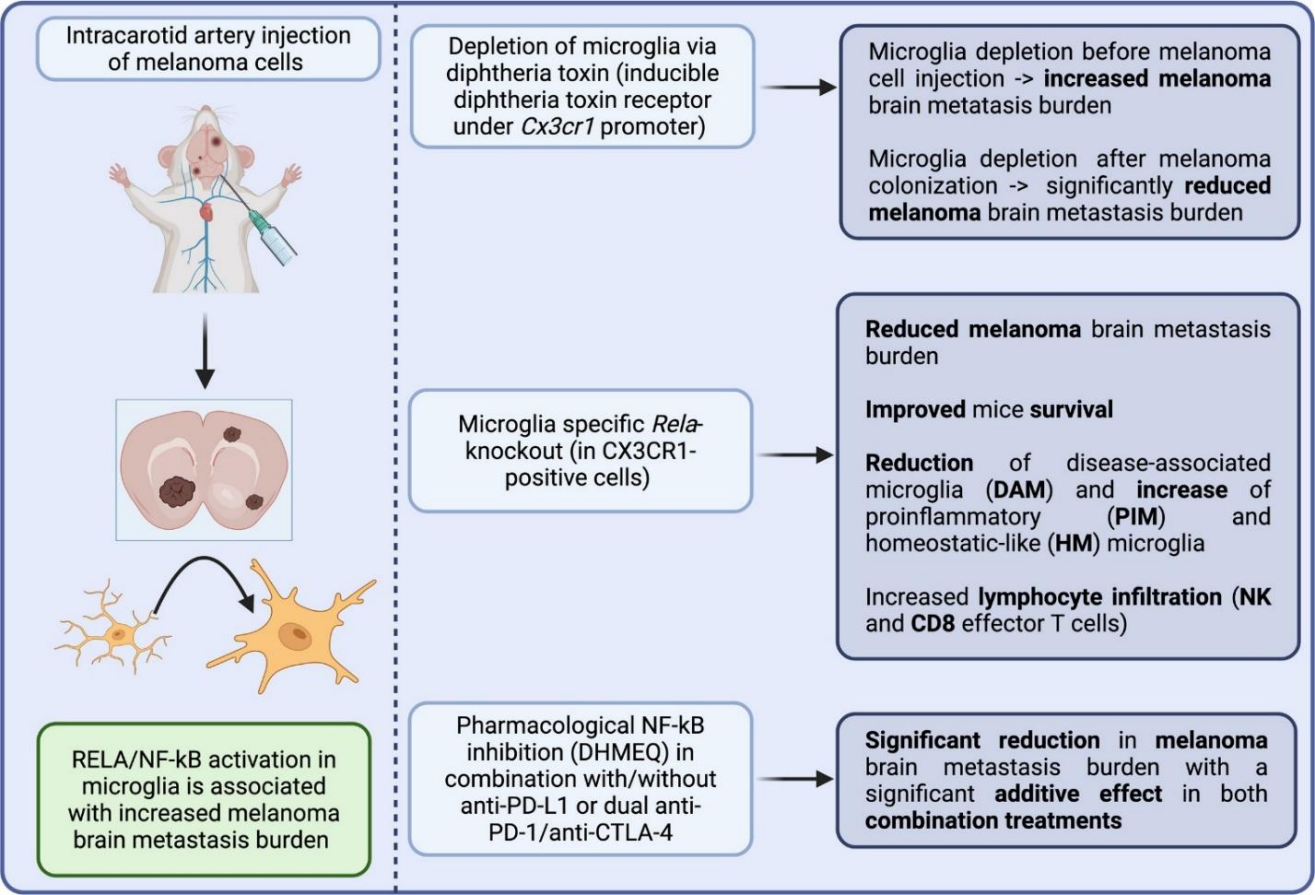


## 3. Divergent vascular and immune landscapes in gliomas and brain metastases [Bejarano et al., 2025]

**Figure 3: Integrated transcriptomic and spatial characterization of the vascular landscape across primary and metastatic brain tumors:**
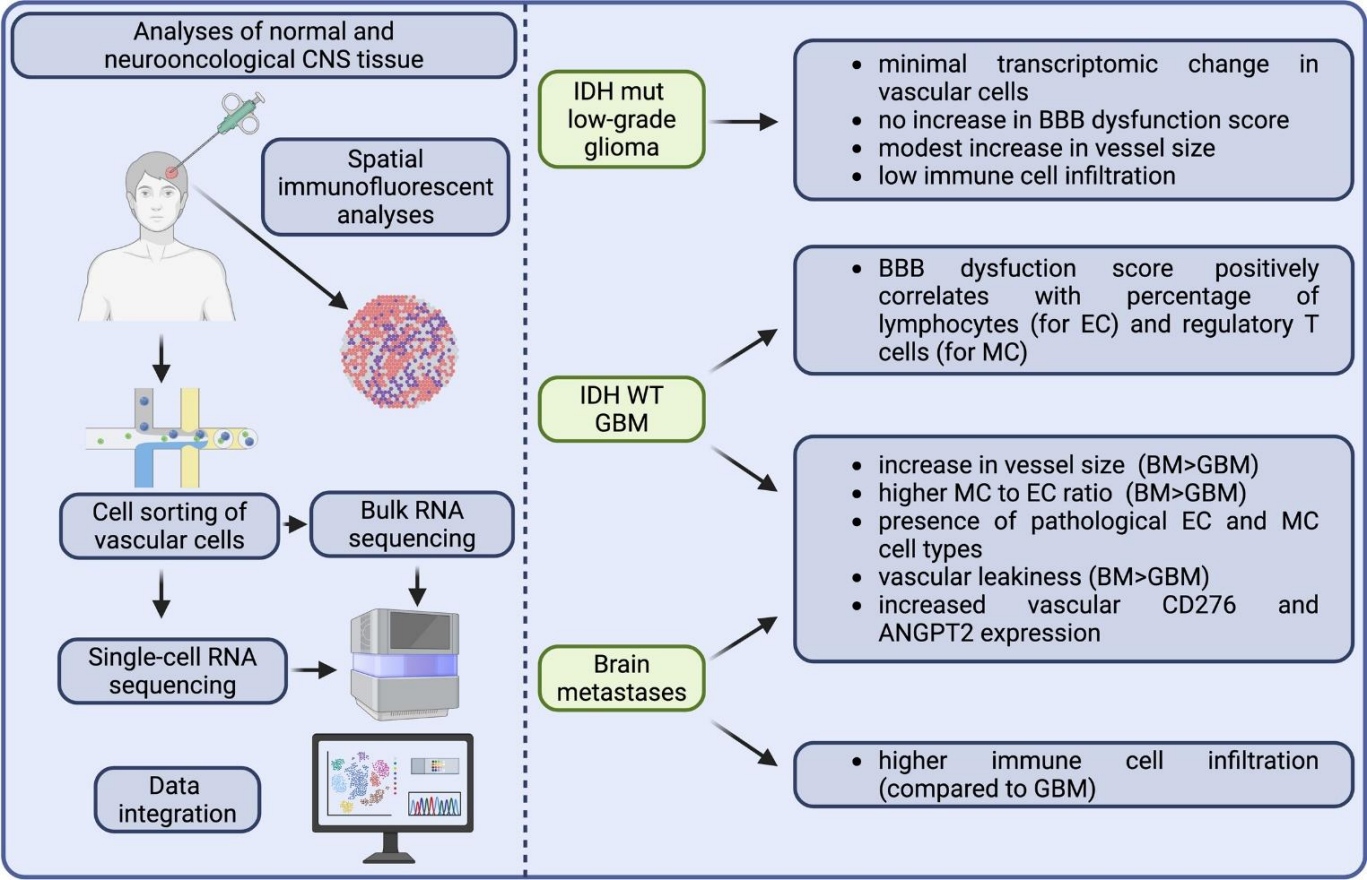
BM: brain metastasis; mut: mutant; GBM: glioblastoma; EC: endothelial cells; MC: mural cells.

The tumor vasculature is an active regulator of immune cell infiltration and therapeutic access in brain tumors, yet comparative analyses across the major CNS tumor entities have been lacking. Bejarano et al. now fill this gap by generating a comprehensive atlas of endothelial and mural cells across non-tumor brain, IDH mutant (IDH mut) low-grade gliomas, IDH wild-type (IDH WT) glioblastomas (GBMs), and brain metastases (BrMs) of lung, breast, and melanoma origin **[Bejarano et al., 2025]**. Using FACS-sorted CD31⁺ and PDGFRβ⁺ cells from freshly resected human tissue, the authors combined bulk and single-cell RNA sequencing (scRNA-seq) with spatial immunofluorescence and cell-cell communication analysis (CellChat), delivering a multi-layered vascular map with direct translational implications **(Figure 3)**. The most striking finding is the near-complete transcriptional quiescence of the vasculature in IDH mut low-grade gliomas, which clustered alongside non-tumor brain controls with only one differentially expressed endothelial gene. This stands in sharp contrast to IDH WT GBMs (518 differentially regulated endothelial genes) and BrMs (1294 differentially regulated endothelial genes), with shared alterations converging on extracellular matrix (ECM) remodeling, angiogenesis, and leukocyte adhesion as well as shared downregulation of blood-brain barrier (BBB) maintenance, transport, and neurotransmission genes. Structurally, vessel size, mural cell-to-endothelial cell ratio, extravascular fibrinogen, and collagen-IV deposition all followed the same hierarchy: non-tumor similar to IDH mut < IDH WT GBM < BrM. These vascular parameters correlated meaningfully with immune cell infiltration: fibrinogen associated with myeloid populations, while collagen IV correlated with perivascular lymphocyte retention, suggesting that ECM remodeling actively shapes immune cell positioning rather than merely reflecting vascular injury. Single-cell resolution revealed seven endothelial subtypes (including angiogenic, interferon, and proliferative populations) and seven mural cell subtypes (transport, ECM, interferon, and proliferative pericytes, two smooth muscle cell types, and fibroblasts). IDH WT GBMs were dominated by ECM pericytes (> 62 %) and angiogenic endothelial cells, while BrMs additionally harbored interferon and proliferative clusters, an immune-activated signature consistent with their higher lymphocyte infiltration and supporting the notion that combining vascular-targeting with immunotherapy may be more effective in BrMs than in GBMs. Both tumor types shared vascular overexpression of the immune checkpoint molecule CD276 (B7-H3), validated by spatial immunofluorescence, and showed convergent upregulation of galectin signaling. These represent actionable shared targets, with CD276-blocking approaches and dual ANGPT2/VEGF inhibition already showing efficacy in preclinical models **[Dai et al., 2025; Di Tacchio et al., 2019]**. The parallel isolation of both endothelial and mural cells, the latter chronically understudied, from the same specimens is a genuine methodological advance, and the integration across bulk sequencing, scRNA-seq, and spatial analyses, validated against two independent public glioma datasets, is commendable. Several limitations deserve acknowledgment, however. The non-tumor controls derive from epilepsy patients who are significantly younger than the tumor cohorts; while covariate analyses found no age-driven confounding, biological differences in vascular aging cannot be excluded. The scRNA-seq component was restricted to four glioma patients, limiting inter-patient heterogeneity capture, and reliance on PDGFRβ as the sole mural cell marker may underrepresent distinct mural cell populations. Cell-cell communication inference via CellChat is probabilistic and unvalidated spatially for the key hits identified. The comparison with neurological disorders is conceptually interesting but methodologically inconsistent, as external datasets used single-nuclei rather than single-cell sequencing. Finally, while CD276, ANGPT2, and galectin pathways are compellingly nominated as therapeutic targets, functional vascular-specific evidence in human tumors remains to be established. These caveats notwithstanding, Bejarano et al. provide a landmark resource that should serve as a key reference for the neurooncology field and a foundation for designing rational vascular- and immune-targeting combination strategies.

## 4. Context-dependent pro-tumorigenic functions of ZIC1 in medulloblastoma [Lee et al., 2025]

**Figure 4: Context-dependent oncogenic functions of ZIC1 in medulloblastoma:**
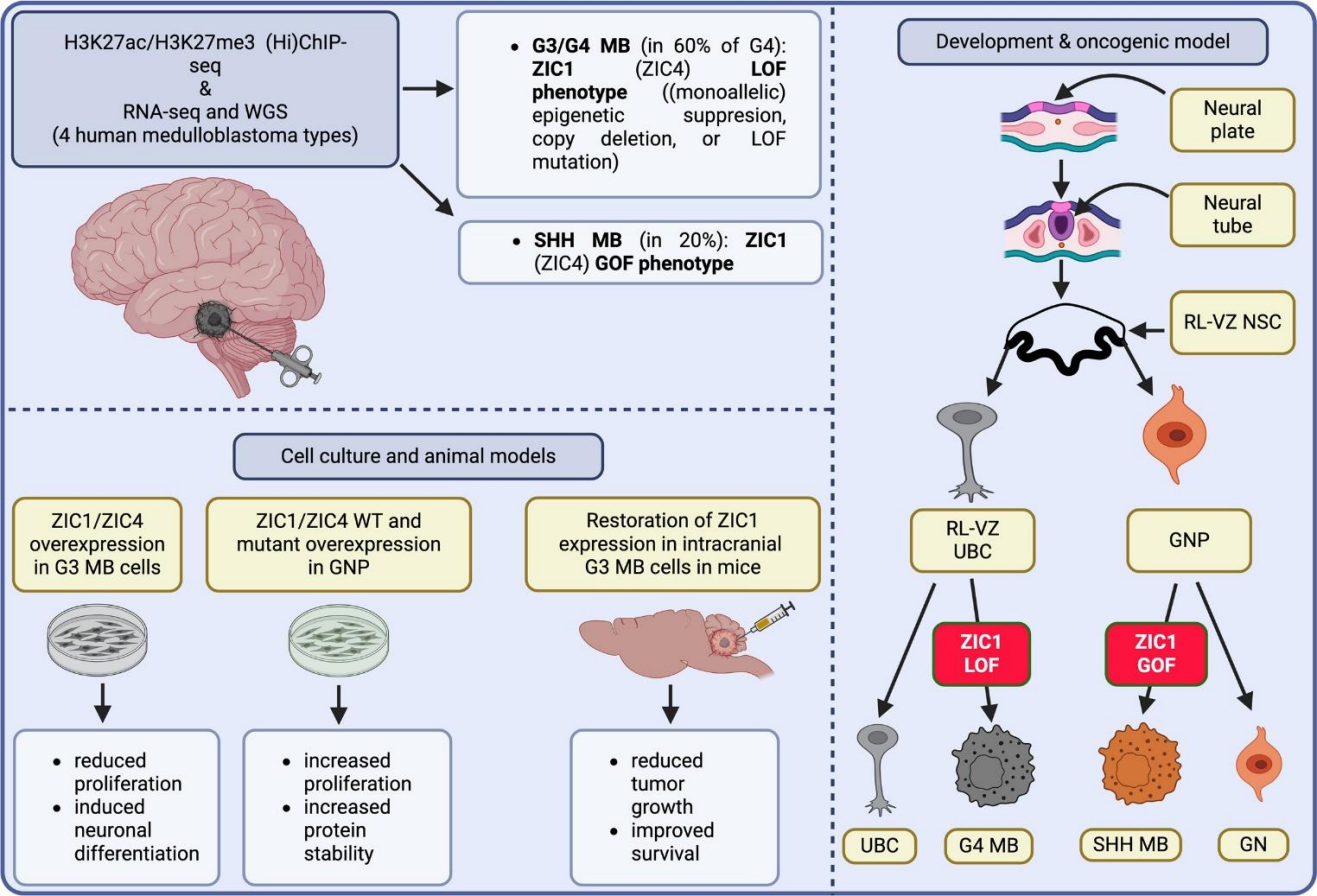
H3K27ac = Histone H3 lysine 27 acetylation; H3K27me3 = Histone H3 lysine 27 trimethylation; (Hi)ChIP-seq = (High-throughput) chromatin immunoprecipitation sequencing; RNA-seq = RNA sequencing; WGS = Whole genome sequencing; MB = Medulloblastoma; G3/G4 MB = Group 3/Group 4 medulloblastoma; SHH MB = Sonic hedgehog medulloblastoma; ZIC1/ZIC4 = Zinc finger protein of the cerebellum 1/4; LOF = Loss of function; GOF = Gain of function; WT = Wild-type; GNP = Granule neuron precursor; RL-VZ NSC = Rhombic lip ventricular zone neural stem cell; RL-VZ UBC = Rhombic lip ventricular zone unipolar brush cell; UBC = Unipolar brush cell; GN = Granule neuron.

The study by Lee and colleagues identifies ZIC1 as a remarkably context-dependent pro-tumorigenic driver gene in medulloblastoma. Using integrated chromatin profiling, sequencing approaches, and functional analyses, the authors demonstrate that ZIC1 undergoes loss-of-function alterations in group 4 (G4) medulloblastoma, while the same gene exhibits gain-of-function characteristics in Sonic hedgehog-activated (SHH) medulloblastoma. A major strength of the work is the elegant integration of epigenetics and developmental neurobiology. The authors identify an unusual "H3K27ac–H3K27me3 hemizygous state" at the *ZIC1/ZIC4* super-enhancer locus in a substantial subset of G4 tumors, resulting in monoallelic repression and reduced transcript expression. Together with recurrent deletions and zinc-finger domain mutations, these findings support the concept that ZIC1 acts as a loss-of-function driver in G4 medulloblastoma. In contrast, SHH tumors displayed copy-number gains and mutations clustering within the C-terminal region of *ZIC1*, consistent with gain-of-function activity. Functionally, ZIC1 overexpression suppressed proliferation in group 3 medulloblastoma models, whereas SHH precursor systems exhibited enhanced proliferative activity **(Figure 4)**. These opposing biological effects strongly support the notion that transcription factor activity in pediatric brain tumors is highly lineage dependent. Particularly compelling is the developmental context of the findings, as ZIC1 and ZIC4 are well-established regulators of rhombic lip and cerebellar development and are implicated in Dandy–Walker malformation **[Grinberg et al., 2004]**. The study therefore links developmental dysregulation with subgroup-specific oncogenesis in an impressive manner. However, functional validation was partly restricted by the absence of faithful G4 model systems, necessitating the use of group 3 cell lines in several experiments. In addition, the mechanisms leading to aberrant H3K27me3 deposition remain largely unresolved. While chromatin modifier mutations may contribute to this phenotype, causality has not yet been established. Overall, this study represents an important conceptual advance in medulloblastoma biology. It demonstrates that identical developmental transcription factors may exert diametrically opposed oncogenic functions depending on cellular lineage and (epi-)genetic context. Beyond ZIC1 itself, the work highlights how tightly developmental programs and pediatric brain tumorigenesis are interconnected.

## 5. Prognostic immune-hot proteomic subtypes in IDH-mutant glioma [Tang et al., 2025]

**Figure 5: Multi-omics stratification of IDH-mutant astrocytoma revealing four prognostically relevant proteomic tumor subtypes:**
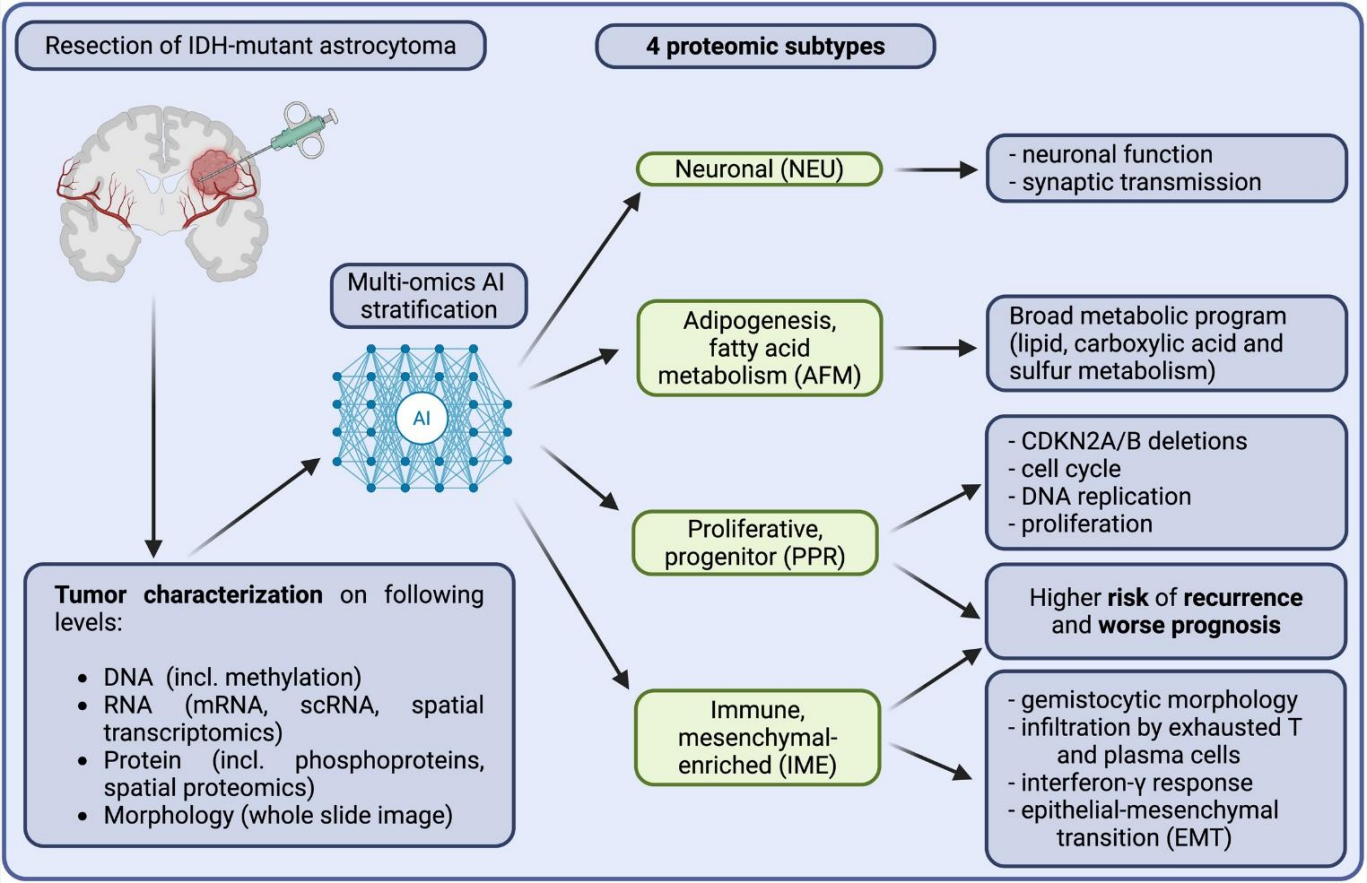


IDH-mutant astrocytoma remains lethal despite improved molecular diagnostics, with intertumoral heterogeneity poorly captured by genomics and transcriptomics alone. Tang et al. assembled a spatiotemporal multi-omics cohort of 51 IDH-mutant astrocytomas with two independent validation cohorts (The Cancer Genome Atlas (TCGA), n = 234; Chinese Glioma Genome Atlas (CGGA), n = 273), alongside spatial proteomics and transcriptomics platforms **[Tang et al., 2025]**. Unsupervised non-negative matrix factorization (NMF) of proteomic data identified four subtypes: adipogenesis/fatty-acid-metabolism (AFM), proliferative/progenitor (PPR), immune/mesenchymal-enriched (IME), and neuronal (NEU). While PPR's poor prognosis is explained by CDKN2A/B deletion enrichment and WHO grade 4 enrichment, the IME subtype, comprising approximately 13 % of tumors across cohorts, showed equivalently poor overall and progression-free survival without these markers, remaining an independent predictor of adverse outcome, warranting consideration in future WHO classification refinements. Gemistocytic differentiation (GD), tumor cells with enlarged eosinophilic cytoplasm and eccentric nuclei, proved the defining histomorphological hallmark of IME. An AI-powered whole-slide imaging GD classifier achieved a similarly high performance as compared to neuropathologist scorings. Single-cell RNA sequencing and spatial proteomics revealed an immune-hot tumor microenvironment with exhausted CD8+ T cells, enriched plasma cells, and perivascular lymphocytic cuffing, paradoxically associating with poor prognosis. Mechanistically, upregulation of the interferon-inducible immune genes GBP1/GBP2 and the serine protease inhibitor SERPINA3 in GD tumor cells likely promotes blood-brain barrier disruption and plasma cell infiltration, with IgG-mediated tumor cell engagement via FCGR2A (Fc gamma receptor IIA). GBP1 overexpression increased proliferation and migration in an IDH-mutant cell line *in vitro*. Longitudinally, recurrent tumors exhibit a reduced proportion of GD cells, which are outnumbered by aligned fibroblast-like cells ("oncostreams"), whereas the IME molecular signature remains largely conserved. This discordance renders reliance on histomorphology alone a potential diagnostic pitfall in the assessment of recurrence. The AI multi-omics classifier GUIDE integrates imageomics, proteomics, transcriptomics, and methylation, achieving approximately 0.84 accuracy from imaging alone with robust performance. The discovery cohort of 51 patients is small thereby limiting mechanistic conclusions. GBP1 functional evidence derives from a single cell line, and the proposed plasma cell-BBB-GD axis remains correlative. In addition, GUIDE's external validation comprised only 47 patients. Another potential issue is whether multiple proteomic phenotypes co-occur within a single sample and whether this renders proteomics inferior to methylation-based diagnostics. Spatial proteomics confirmed layered microanatomical niches in IME tumors, meaning bulk proteomics suffers a sampling-dependent heterogeneity. Proteomics adds genuine information as protein subtypes are not recoverable by transcriptomics alone, with IME-specific extracellular proteins showing markedly attenuated mRNA versus protein fold changes. But unlike methylation profiling, which is standardized across thousands of CNS reference samples and embedded in neuropathological guidelines **[Capper et al., 2018]**, proteomics lacks the inter-institutional pre-analytical standardization for routine diagnostics. The modalities are complementary rather than competing, with methylation currently retaining a decisive practical advantage for routine clinical implementation. However, it is a testament to the enduring relevance of classical neuropathology that gemistocytic differentiation, described long before the molecular era, now reemerges as a morphological hallmark linking spatial immune architecture, proteomic identity, and clinical outcome in the age of multi-omics analyses **(Figure 5)**.

## 6. Opening the black box of brain tumor AI diagnostics [Benfatto et al., 2025]

DNA methylation profiling has become an essential component of modern neuropathological diagnostics, particularly through the Heidelberg brain tumor classifier. While this machine learning-based approach achieves highly accurate classification of CNS tumors, its underlying decision-making process has remained largely inaccessible. In their study, Benfatto et al. introduce an explainable AI (XAI) framework designed to uncover how the classifier distinguishes between more than 80 tumor entities **[Benfatto et al., 2025]**. The authors analyze the random forest architecture of the Heidelberg classifier by systematically extracting and quantifying the usage of methylation probes within more than 10,000 decision trees. Their analyses demonstrate that only a relatively small subset of probes contributes substantially to tumor classification, while most probes show minimal relevance. Importantly, the study reveals that the classifier recognizes biologically meaningful genomic structures, including CpG islands, enhancer regions, and heterochromatic domains, rather than relying on arbitrary statistical patterns. For example, IDH-mutant gliomas were mainly identified through hypermethylated CpG island probes, reflecting the known CpG island methylator phenotype. A major strength of the work lies in its biological interpretation of machine learning decisions. The authors demonstrate that multiple genes and genomic regions redundantly contribute to tumor classification, thereby increasing classifier robustness. From a translational perspective, these findings may facilitate the development of smaller and more practical diagnostic methylation panels. Furthermore, the publicly accessible web application significantly improves transparency and allows researchers to explore class-specific methylation signatures (see https://hovestadtlab.shinyapps.io/shinyMNP/). Nevertheless, several limitations remain. The study is primarily descriptive and does not establish direct biological causality for the identified methylation patterns. In addition, the explainability framework is closely linked to the random forest architecture of the Heidelberg classifier and may not be directly transferable to other AI systems such as deep learning models. Although the framework improves interpretability, the resulting multidimensional datasets remain highly complex and may still be difficult to integrate into routine clinical diagnostics. In summary, Benfatto et al. provide an important contribution to XAI in neurooncology. The study demonstrates that methylation-based classifiers capture biologically relevant epigenetic structures and represents a meaningful step toward improving transparency, clinical trust, and future biomarker discovery in AI-assisted brain tumor diagnostics.

## 7. Real-time epigenetic brain tumor classification with sparse nanopore data [Brändl et al., 2025]

Brändl and colleagues present MethyLYZR, a probabilistic classifier for rapid intraoperative CNS tumor diagnosis using sparse nanopore methylation data **[Brändl et al., 2025]**. The study addresses a central problem in modern neuropathology: although methylation-based tumor classification has become diagnostically indispensable, current workflows require days rather than the approximately one-hour window during which neurosurgical decisions are made. The key conceptual contribution is surprisingly simple: instead of relying on computationally demanding deep-learning systems or sample-specific retraining approaches, the authors apply a weighted Bernoulli naïve Bayes classifier to sparse methylation signals generated by shallow nanopore sequencing. This approach is particularly suited to intraoperative sequencing because most CpG sites are missing at random; unlike many machine-learning models, naïve Bayes can largely ignore such missingness without collapsing diagnostically. Using the established Heidelberg/DKFZ methylation reference cohort, the authors trained the model on 91 CNS methylation classes, later condensed into 44 clinically relevant groups. Synthetic sparse datasets demonstrated approximately 95 % accuracy with only 7,500 CpGs, a quantity achievable within roughly 15 minutes of sequencing. Importantly, most classification errors remained within related diagnostic families rather than across biologically unrelated tumors. The technical workflow is one of the study’s major strengths. DNA extraction (∼22 min), rapid library preparation (∼18 min), and sequencing (∼15 min) enabled a complete biopsy-to-result pipeline within one hour. Computational demands were minimal, with prediction runtimes below one second for clinically relevant datasets. In contrast to neural-network approaches such as Sturgeon, MethyLYZR avoids extensive retraining and heavy hardware requirements while still outperforming competing methods under sparse-data conditions **[Vermeulen et al., 2023]**. Clinical validation was promising, as across 75 nanopore sequencing runs from 51 patients, the model achieved 94.5 % accuracy for clinically-grouped CNS classes using only the first 15 minutes of sequencing data. Concordance with EPIC methylation arrays was complete in the tested subset. The classifier also showed feasibility for diagnoses beyond primary CNS tumors, including brain metastases, multiplexed sequencing workflows, and cerebrospinal fluid (CSF)-derived cell-free DNA (cfDNA) samples, suggesting broader applicability beyond intraoperative tissue diagnostics. Several limitations temper the enthusiasm as the approach still depends heavily on historical methylation-array reference datasets because large public sequencing-based methylation atlases remain scarce. Second, diagnostic performance strongly depended on tumor purity, with reliable predictions mainly achieved above 60–70 % tumor content. This represents a major unresolved issue for infiltrative gliomas, margin assessment, and treatment-effect specimens, the settings where intraoperative molecular diagnostics would be most valuable. The study also raises a broader methodological point as MethyLYZR’s success challenges the assumption that increasingly complex AI systems necessarily outperform simpler statistical models in clinical medicine. Under conditions of sparse and noisy intraoperative data, the framework of this study proved not only faster and more interpretable, but also more accurate than more sophisticated alternatives. Overall, this study represents an important translational advance in molecular neuropathology. MethyLYZR does not solve all limitations of intraoperative methylation profiling, but it convincingly demonstrates that clinically meaningful epigenetic tumor classification within surgical time constraints is technically feasible. Whether this approach will become part of routine neuropathological workflows now depends less on algorithmic innovation than on prospective multicenter validation and integration into real-world surgical practice.

## 8. Nanopore sequencing enters the operating room: rapid molecular profiling of CNS tumors [Patel et al., 2025]

Comprehensive molecular profiling is now mandatory for WHO-compatible CNS tumor diagnosis, yet conventional array-based workflows are costly, labor-intensive, and routinely require days to weeks, effectively confining precision diagnostics to high-throughput academic centers. Patel et al. address this gap by comprehensively validating Rapid-CNS^2^, an adaptive-sampling nanopore sequencing pipeline, in a prospective multicenter setting across 301 archival and prospective samples from University Hospital Heidelberg and the University of Nottingham, including 18 samples sequenced intraoperatively. Complementing this, the authors introduce MNP-Flex, a gradient-boosted, platform-agnostic methylation classifier covering all 184 MNP v.12 subclasses, validated on a global cohort of over 78,000 samples spanning five sequencing technologies **[Patel et al., 2025]**. The pipeline demonstrated a mean turnaround time of approximately 30–40 hours from tissue receipt to complete report, compared to several weeks conventionally, covering single nucleotide variants (SNVs), copy number variants (CNVs), gene fusions, *MGMT* promoter methylation, and methylation classification in a single workflow. Despite these highly encouraging results, nanopore sequencing still exhibits inherent technical limitations that warrant caution before widespread clinical implementation. Systematic sequencing errors, context-dependent base-calling inaccuracies, and challenges in homopolymeric or repetitive regions remain recognized features of the technology, although they have been substantially reduced with newer chemistries and computational approaches. These limitations may affect variant detection and molecular classification performance and therefore require continued validation against established diagnostic platforms **[Delahaye and Nicolas, 2021; Liu-Wei et al., 2024]**. In the study by Patel et al., SNV recovery against matched next-generation sequencing (NGS) panel data reached 91.67 %, with *IDH1/2* and *BRAF* mutation calls correct in 47 of 48 cases (97.9 % sensitivity, 100 % specificity) **[Patel et al., 2025]**. Copy number profiles showed complete concordance with methylation array counterparts across all 254 matched samples. At the methylation family level, 251 of 270 classifiable cases were correctly assigned (92.9 %), rising to 96.1 % with a 30 % confidence filter applied. Integrated diagnoses were concordant with conventional reference in 285 of 301 cases (94.6 %), with potentially misleading results in only 5 cases (1.6 %) — a rate consistent with established array-based classifiers. Notably, all small biopsy, recurrent tumor, and infiltration zone samples yielded concordant integrated diagnoses, underscoring the pipeline's robustness across challenging specimen types. Intraoperatively, 29 of 35 samples with sufficient reads were correctly classified within 15 minutes, and arm-level CNVs, sufficient to distinguish IDH mutant astrocytoma from IDH wild-type glioblastoma, were resolved after only 10 minutes, two entities otherwise indistinguishable on frozen section morphology alone. In prospective real-time runs, clinically relevant molecular information was available within 30 minutes in 13 of 18 cases (72.2 %). One illustrative case initially suspected as glioma was reclassified within 30 minutes as a CIC-altered Ewing family tumor, confirmed by methylation array five days later versus one month by conventional methods. MNP-Flex, validated across whole genome bisulfite sequencing (WGBS), Oxford Nanopore Technologies (ONT) whole genome sequencing (ONT-WGS), Twist panels, and Rapid-CNS^2^ data from seven global institutions, achieved 99.6 % family-level and 99.2 % subclass-level accuracy with applicable confidence thresholds, enabling reclassification of cases falling outside the v.11 classifier scope into newly defined v.12 entities. While the study's multicenter prospective design and breadth of validation are commendable, several limitations merit critical attention. Most consequentially, Rapid-CNS^2^ remains restricted to fresh or cryopreserved tissue, as FFPE-derived short DNA fragments are incompatible with adaptive sampling, a significant barrier given that FFPE is the dominant tissue type in routine neuropathology worldwide. The intentional inclusion of 31 cases not resolvable by methylation (e.g., brain metastases) without censoring them complicates straightforward interpretation of the error rate. The elevated CpG missingness in nanopore data (∼16.6 % versus < 0.7 % in other methods) systematically suppresses MNP-Flex confidence scores, suggesting classifier performance in this modality could be further optimized through missingness-aware training. Critically, none of the intraoperative results were yet used to guide surgical decision-making, and the anticipated prospective outcome study remains pending, an essential step before clinical implementation can be responsibly recommended. Finally, while infrastructure cost advantages over array-based platforms are emphasized throughout, the practical requirements of graphics processing unit (GPU) hardware for base-calling and device consumables deserve more transparent discussion, particularly for the resource-limited settings this technology aspires to serve. Despite these caveats, the combined Rapid-CNS^2^/MNP-Flex framework represents a genuinely transformative step toward democratizing comprehensive molecular CNS tumor diagnostics on a global scale.

## 9. Real-time AI-guided detection of glioma surgical margins using label-free optical microscopy and a self-supervised foundation model [Kondepudi et al., 2025]

**Figure 6:**
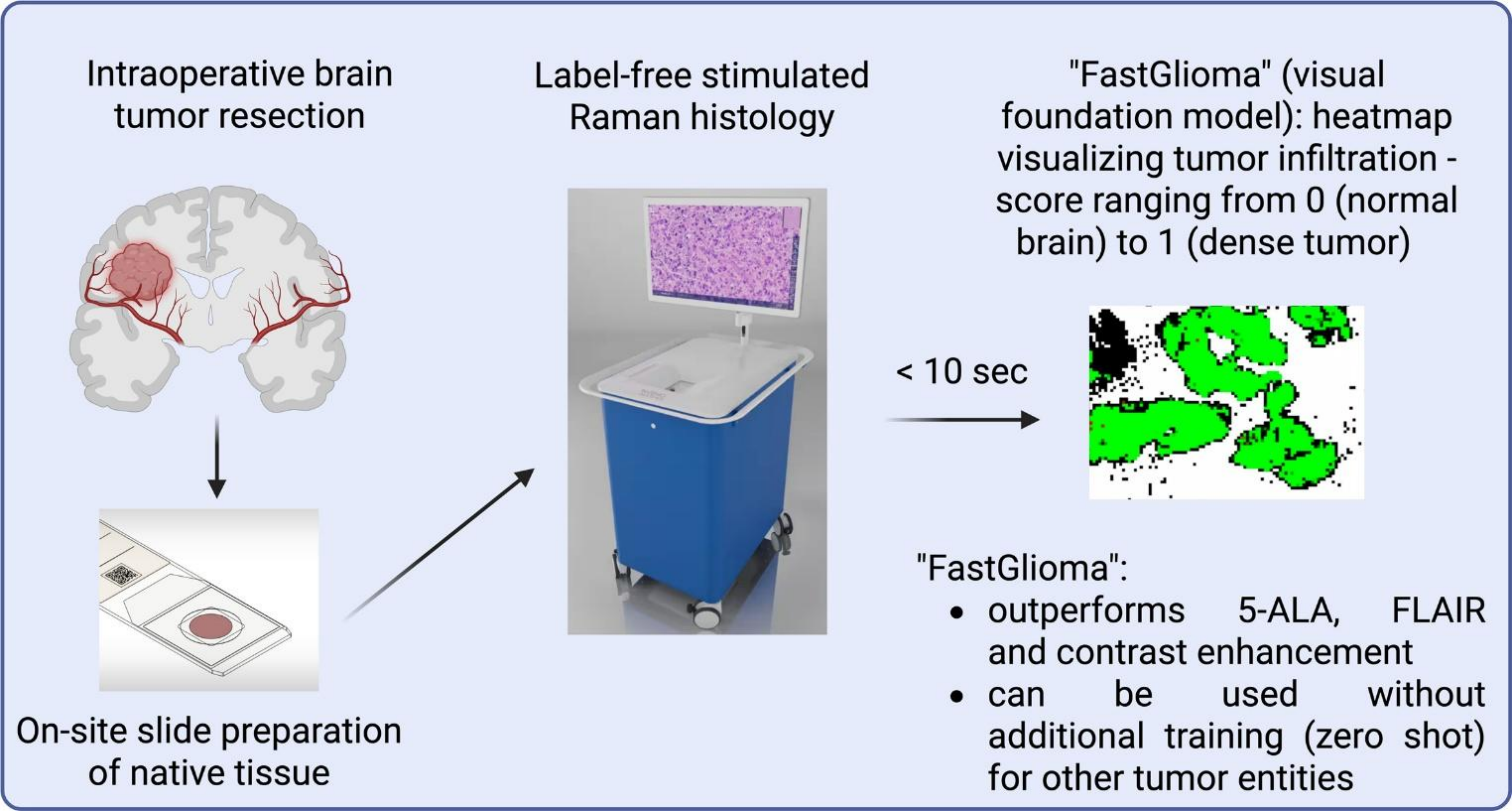


There is growing evidence that supramaximal resection in primary brain tumors may be associated with improved patient survival, underscoring the need for a clear and unequivocal definition of intraoperative surgical margins.** [Ambati and Hervey-Jumper, 2025]**. To overcome classic histological, time-consuming intraoperative diagnostics, stimulated Raman scattering (SRS) microscopy has been previously implemented **[Orringer et al., 2017]**. Based on this approach, the study of Kondepudi et al. introduces *FastGlioma*, an open-source foundation model-based framework for rapid, label-free intraoperative detection of glioma infiltration, addressing the reliable identification of tumor margins in real time **[Kondepudi et al., 2025]**. The authors combine stimulated Raman histology (SRH) with large-scale self-supervised learning to produce quantitative infiltration scores within seconds. The two-stage visual foundation model is trained on approximately four million SRH patches from more than 11,000 surgical specimens. Fine-tuning employs ordinal metric learning, mapping histological patterns onto a continuous infiltration axis from neuropathologist-defined categories, elegantly addressing annotation scarcity by exploiting weak, slide-level labels while retaining spatial interpretability. Unlike frozen-section histopathology, SRH enables near real-time imaging without staining. Fast-acquisition SRH, requiring approximately 10 seconds, yields nearly equivalent performance to higher-resolution scans, supporting intraoperative feasibility, though reliance on specialized hardware may limit scalability in resource-constrained settings. The prospective multicenter validation across 220 patients and 1,500+ specimens outperformed supervised models and standard adjuncts including MRI neuronavigation and 5-aminolevulinic acid (5-ALA) fluorescence. Robustness across glioma subtypes, molecular classes, and clinical sites suggests biologically relevant feature representations. The simulated interventional analysis showed *FastGlioma* markedly reduced high-risk false-negatives versus standard-of-care (3.8 % vs. 24 %), though actual impact on outcomes requires prospective randomized validation. Interpretability is addressed through few-shot visualizations comparing query regions to clinician-defined support sets, generating intuitive infiltration heatmaps without retraining. However, reliance on manually selected exemplars introduces subjectivity requiring further investigation. Key limitations include restriction to SRH imaging, not yet widely adopted clinically, oversimplification of tumor-brain interfaces through ordinal scoring, and inadequate assessment of biases from patient selection or institutional practices. Furthermore, while a zero-shot approach for non-glioma entities may be effective in telencephalic regions with conventional gray and white matter architecture, its applicability to brain areas with distinct neuroanatomical organization, such as the brainstem or cerebellum, remains to be determined. Overall, *FastGlioma* represents a significant advancement at the intersection of neurooncology, optical imaging, and AI, but its true impact depends on clinical workflow integration, interventional trial validation, and broader imaging technology accessibility **(Figure 6)**.

## 10. Patient-derived brain tumor organoids: toward functional precision neurooncology [Peng et al., 2025]

**Figure 7: Workflow of individualized patient tumor organoid (IPTO) model generation:**
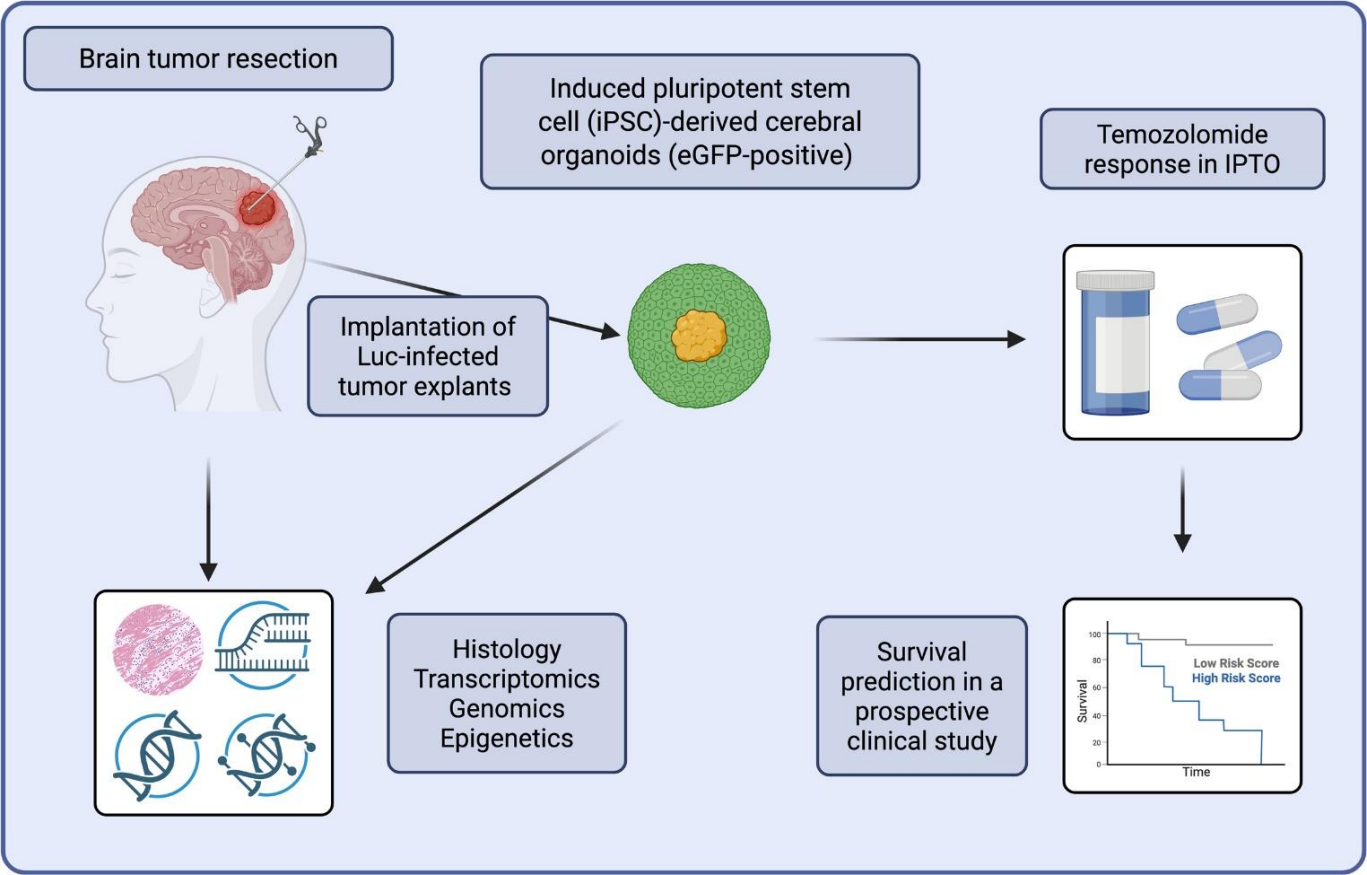
iPSC: induced pluripotent stem cells; Luc: luciferase.

The study by Peng et al. introduces an individualized patient tumor organoid (IPTO) model that represents a significant advance in translational neurooncology. By integrating freshly resected tumor explants into induced pluripotent stem cell-derived cerebral organoids, the authors generated cultures that preserve the histological, molecular, and cellular characteristics of a broad spectrum of CNS tumors, including glioblastoma, lower-grade glioma, pediatric tumors, and brain metastases **[Peng et al., 2025]**. A major strength of the work is the preservation of intratumoral heterogeneity and microenvironmental complexity. IPTOs retained proliferative activity, tumor-associated macrophages, vascular structures, and regional differences between tumor core and rim. Compared to previous organoid systems such as patient-derived GBM organoid (GBO; with or without Matrigel) or cerebral organoid glioma (GLICO; patient-derived dissociated cells or spheres), the IPTO model demonstrated markedly improved maintenance of immune and stromal components. This is highly relevant, as glioblastoma biology is strongly shaped by its microenvironment. Equally convincing are the molecular analyses: whole-exome sequencing, DNA methylation profiling, and single-cell RNA sequencing revealed high concordance between organoids and parental tumors. Importantly, clinically relevant molecular subclasses and epigenetic signatures were preserved even during long-term culture. Such stability contrasts with conventional cell culture models, which frequently undergo molecular drift. The most translationally relevant aspect of the study is the demonstration that IPTOs may predict patient-specific therapeutic responses. Temozolomide sensitivity in organoids correlated with clinical outcome, suggesting that the model could serve as a functional platform for personalized therapy testing. Despite these impressive findings, several limitations remain, namely cerebral organoids resemble immature developmental brain tissue rather than the aged CNS environment in which glioblastoma typically arises. Thus, important age-related stromal and immune interactions may not be fully represented. Furthermore, although immune cells are preserved better than in earlier models, systemic immune responses and complex immunotherapeutic mechanisms cannot be adequately recapitulated. Practical implementation into clinical workflows also remains challenging. Standardization, reproducibility, costs, and turnaround times suitable for real-time treatment decisions still need validation in larger prospective studies. Moreover, predictive value has thus far mainly been demonstrated for temozolomide, whereas modern neurooncology increasingly relies on multimodal treatment strategies. In summary, Peng et al. provide a highly sophisticated and biologically faithful brain tumor organoid platform with considerable translational potential. Although technical and biological limitations remain, the IPTO model constitutes an important step toward functional precision medicine in neurooncology **(Figure 7)**.

## Discussion

The studies highlighted in this year’s "top ten" series illustrate that neurooncology is currently undergoing a paradoxical phase of unprecedented technological acceleration while simultaneously facing profound translational therapeutic stagnation. Rarely before have molecular diagnostics, spatial transcriptomics, proteomics, AI, nanopore sequencing, or real-time intraoperative technologies evolved with such remarkable speed. Yet, despite this enormous methodological progress, the overall prognosis for patients suffering from highly malignant CNS tumors remains devastatingly poor in routine clinical reality. Glioblastoma especially continues to behave as one of the most unforgiving human malignancies, with population-based survival times still frequently below one year even in highly developed healthcare systems. Several of the discussed studies further reinforce an increasingly uncomfortable concept: glioblastoma is not merely a genetically aberrant neoplasm but rather a highly adaptive organ-like ecosystem capable of exploiting virtually every physiological system of the host brain. Neuron-glioma synaptic integration, vascular remodeling, immune microenvironment reprogramming, and metabolic plasticity all converge toward one central hallmark: the extraordinary evolutionary adaptability of malignant CNS tumors **[Bejarano et al., 2025; Rodriguez-Baena et al., 2025; Tang et al., 2025; Tetzlaff et al., 2025]**. Particularly, the findings on glioma-neuron interactions may force neurooncology to reconsider long-established dogmas **[Tetzlaff et al., 2025]**. If neuronal activity itself promotes glioma progression and therapeutic resistance, the classical separation between "tumor biology" and "normal brain physiology" becomes increasingly artificial. In this regard, glioblastoma may not simply invade the brain but progressively function as a distorted neurobiological component of it. At the same time, the field is currently experiencing a nearly euphoric expansion of AI applications. AI-supported intraoperative diagnostics, explainable methylation classifiers, and foundation models for surgical margin detection undoubtedly represent highly impressive achievements **[Benfatto et al., 2025; Brändl et al., 2025; Kondepudi et al., 2025; Patel et al., 2025]**. Nevertheless, one should remain cautious not to confuse diagnostic sophistication with therapeutic progress. Neurooncology has historically suffered from waves of technological enthusiasm that ultimately failed to significantly alter patient survival. It is therefore legitimate to ask whether parts of modern neurooncology are at risk of becoming increasingly "diagnostic-rich but therapy-poor". The capacity to molecularly characterize a fatal disease within minutes is scientifically fascinating; however, for many patients worldwide this still does not translate into substantially prolonged survival or preserved neurological function. This discrepancy becomes even more problematic when viewed in the context of global healthcare inequalities **[Santosh et al., 2026]**. Several technologies discussed in this review, including intraoperative nanopore sequencing, spatial multi-omics, AI-supported imaging or single-cell analyses, require infrastructure, computational resources and interdisciplinary expertise that remain inaccessible to most regions of the world **[Benfatto et al., 2025; Brändl et al., 2025; Kondepudi et al., 2025; Patel et al., 2025]**. While precision neurooncology is rapidly evolving in highly specialized quaternary centers, large parts of the global population still lack access to basic MRI diagnostics, modern radiotherapy, or standardized neuropathological classification. Thus, the field risks entering a scientifically dazzling but ethically uncomfortable era of "hyper-personalized neurooncology for the few". Particularly in low- and middle-income countries, where demographic aging and increasing cancer incidence will likely produce a considerable rise in CNS tumor burden over the coming decades, this imbalance may become even more pronounced **[Chen et al., 2025]**. In parallel, worldwide political developments increasingly influence neurooncological research itself. International scientific collaborations have become more fragile due to geopolitical tensions, economic instability, and growing nationalist tendencies in research funding policies **[Vanino et al., 2026]**. Moreover, global healthcare systems continue to suffer from long-term consequences of the COVID-19 pandemic, workforce shortages, and escalating economic pressure **[Woodward et al., 2025]**. Neurooncology and related neuropathology, disciplines inherently dependent on highly specialized interdisciplinary structures, may therefore become particularly vulnerable to political and financial destabilization. One may even provocatively argue that modern neurooncology currently advances scientifically faster than healthcare systems are capable of implementing its innovations responsibly and equitably. This challenge also calls for a reorientation of research funding priorities. Funding agencies should consider establishing dedicated programs that incentivize the development of affordable and widely deployable diagnostic tools and therapies, while also systematically incorporating cost-effectiveness as an evaluation criterion in translational research. Scientific progress that cannot be implemented at scale risks widening existing disparities in access to care. Therefore, innovation should be judged not only by technical sophistication but also by its feasibility, accessibility, and societal impact. Another important issue concerns scientific reproducibility and publication culture. The immense pressure to publish technologically sophisticated "multi-omics" or AI-driven studies may inadvertently incentivize overstated conclusions, insufficient validation and limited biological interpretability. Especially in the rapidly expanding AI field, there is a danger that methodological novelty may occasionally overshadow clinical relevance. The current enthusiasm surrounding explainable AI and foundation models is certainly justified scientifically, but the field should remain aware that black-box algorithms cannot compensate for insufficient biological understanding or poorly designed clinical trials. Several studies discussed in this review also suggest a possible conceptual shift away from purely tumor-cell-centered approaches. The increasing recognition of vascular niches, skull bone marrow immunity, neuronal circuitry, and microglial plasticity indicates that future neurooncological therapies may increasingly target the broader tumor ecosystem rather than malignant cells alone **[Bejarano et al., 2025; Dobersalske et al., 2024; Rodriguez-Baena et al., 2025; Tetzlaff et al., 2025]**. Especially, the modulation of the CNS immune microenvironment and neuron-tumor interactions may ultimately prove more successful than many previous attempts at directly eliminating glioma cells themselves. Whether such approaches will finally overcome the notorious therapeutic resistance of malignant brain tumors remains uncertain, but they clearly broaden the conceptual landscape of the field. Finally, one of the perhaps most remarkable observations emerging from this year’s studies is the enduring relevance of classical neuropathology. Despite all technological advances, morphological hallmarks such as gemistocytic differentiation, vascular architecture, or spatial tissue organization repeatedly reemerge as biologically meaningful features even within highly sophisticated multi-omics frameworks **[Bejarano et al., 2025; Tang et al., 2025]**. This is an important reminder that modern neurooncology should not abandon histopathology in favor of purely computational approaches but rather integrate both perspectives intelligently. The microscope is certainly no longer sufficient on its own, but neither is AI. In summary, neurooncology in 2025 stands at a fascinating yet uneasy crossroads. The field is generating increasingly detailed molecular, spatial, and computational insights into CNS tumors while still struggling to convert this knowledge into durable therapeutic success for the majority of patients. Future progress will therefore depend not only on technological innovation but equally on global accessibility, biological rigor, interdisciplinary collaboration and clinically meaningful translation. Otherwise, neurooncology risks becoming a discipline capable of describing CNS tumors with extraordinary precision while remaining largely unable to substantially alter their ultimately lethal course.

## Conflict of interest statement

The author declares no conflict of interest.
